# Approaching the Secrets of N-Glycosylation in *Aspergillus fumigatus*: Characterization of the AfOch1 Protein

**DOI:** 10.1371/journal.pone.0015729

**Published:** 2010-12-29

**Authors:** Andrea Kotz, Johannes Wagener, Jakob Engel, Françoise H. Routier, Bernd Echtenacher, Ilse Jacobsen, Jürgen Heesemann, Frank Ebel

**Affiliations:** 1 Max-von-Pettenkofer-Institute, Ludwig-Maximilians-University, Munich, Germany; 2 Department of Cellular Chemistry, Hanover Medical School, Hanover, Germany; 3 Institute for Immunology, University of Regensburg, Regensburg, Germany; 4 Department for Microbial Pathogenicity Mechanisms, Leibniz Institute for Natural Product Research and Infection Biology, Jena, Germany; 5 Faculty of Medicine, Center of Integrated Protein Science Munich, Ludwig-Maximilians-University, Munich, Germany; University of Minnesota, United States of America

## Abstract

The mannosyltransferase Och1 is the key enzyme for synthesis of elaborated protein N-glycans in yeast. In filamentous fungi genes implicated in outer chain formation are present, but their function is unclear. In this study we have analyzed the Och1 protein of *Aspergillus fumigatus*. We provide first evidence that poly-mannosylated N-glycans exist in *A. fumigatus* and that their synthesis requires AfOch1 activity. This implies that AfOch1 plays a similar role as *S. cerevisiae* ScOch1 in the initiation of an N-glycan outer chain. A Δ*afoch1* mutant showed normal growth under standard and various stress conditions including elevated temperature, cell wall and oxidative stress. However, sporulation of this mutant was dramatically reduced in the presence of high calcium concentrations, suggesting that certain proteins engaged in sporulation require N-glycan outer chains to be fully functional. A characteristic feature of AfOch1 and Och1 homologues from other filamentous fungi is a signal peptide that clearly distinguishes them from their yeast counterparts. However, this difference does not appear to have consequences for its localization in the Golgi. Replacing the signal peptide of AfOch1 by a membrane anchor had no impact on its ability to complement the sporulation defect of the Δ*afoch1* strain. The mutant triggered a normal cytokine response in infected murine macrophages, arguing against a role of outer chains as relevant *Aspergillus* pathogen associated molecular patterns. Infection experiments provided no evidence for attenuation in virulence; in fact, according to our data the Δ*afoch1* mutant may even be slightly more virulent than the control strains.

## Introduction

Eukaryotic proteins that enter the secretory pathway in the endoplasmatic reticulum (ER) are glycosylated by distinct sets of enzymes that catalyze either the formation of N- or O-linked glycans. Glycosylation can influence the folding of proteins, their biological activity and half-life. So far, fungal N-glycans have mainly been analyzed in yeast, in particular *Saccharomyces cerevisiae*. The core structures made in the ER are similar in all eukaryotic cells, whereas a remarkable diversity exists in the further processing, branching and elongation of this core structure. In yeasts N-glycans are characterized by their distinct and complex high-mannose structures [1].

The Och1 proteins of *S. cerevisiae* and *C. albicans* are α1,6-mannosyltransferases that initiate a distinct branch in the N-glycan core thereby providing the platform for the subsequent formation of a large poly-mannosylated outer chain [2], [3]. The Δ*och1* mutants of S. *cerevisiae* and *C. albicans* are sensitive to elevated temperatures [2], [3]. The *C. albicans* mutant showed furthermore an attenuated virulence in a murine model of infection [3] and triggered a reduced cytokine response in infected murine macrophages, a phenotype that was linked to recognition of outer chains by the mannose receptor [4].

The N-glycosylation pathway has been studied in detail in *S. cerevisiae* [5]. The early steps take place in the cytoplasm and the ER and are essential and highly conserved between yeasts and filamentous fungi. The so-called high mannose pathway is a cascade of enzymes engaged in the synthesis of elaborated N-glycans. The corresponding genes encoding a set of enzymes reaching from Och1 to Mnn1 in *S. cerevisiae* have been identified in filamentous fungi based on sequence homology [6]. However, data on enzyme activity and N-glycan structures in filamentous fungi are still very limited and provided so far no evidence for the existence of elaborated outer chains.

*Aspergillus fumigatus* is a pathogenic mold that can cause severe, life-threatening infections in immuno-compromised patients [7]. Recognition of this pathogen by the innate immune system is crucial for its successful abatement. Glycostructures are excellent targets for the germ-line encoded pattern recognition receptors (PRRs) of innate immune cells. We have recently characterized two *A. fumigatus* mutants in the mannosyltransferase genes *mnt1* and *mitA*. The Mnt1 protein catalyzes an early and essential step in the formation of O-linked glycans, whereas MitA is required for the synthesis of GDP-mannose:inositol-phosphorylceramide (MIPC)-derived glycosphingolipids. We found no differences in the cytokine response induced in murine macrophages infected with these mutants and their corresponding control strains. This argues against a role of O-linked glycans and MIPC-derived glycosphingolipids as major pathogen-associated molecular patterns (PAMPs) of *A. fumigatus* [8], [9]. In this study we generated a mutant in the *afoch1* gene, encoding the orthologue of the yeast Och1 proteins, and characterized its phenotype. We provide evidence for a mannoyltransferase activity of this protein and its requirement for sporulation under distinct growth conditions.

## Results

### The *A. fumigatus* gene AFUA_5G08580

The *och1* genes of *S. cerevisiae* and *C. albicans* encode α1,6-mannosyltransferases that are essential for the **o**uter **ch**ain elongation of N-linked glycans [2], [3]. A BlastP search in the *A. fumigatus* genome data base using the ScOch1 sequence revealed the highest homology for AFUA_5G08580. This ORF is predicted to contain one intron. PCR amplification from *A. fumigatus* chromosomal and cDNA using primers och1-5′ and och1-3′ resulted in two amplicons of different size. Sequencing of the PCR product derived from cDNA revealed an ORF that differed from the one predicted in the genome entry AFUA_5G08580. (The sequence of the full length mRNA has been submitted to the EMBL data base: Accession number FR667640.) The fact that the mRNA was amplified using a primer localized 173 bp down-stream of the STOP codon indicates the presence of a longer untranslated 3′-region, similar to the one predicted for the homologous *A. nidulans* gene AN4716.4-T.

Using the corrected protein sequence AfOch1 shows 33.7% identity (43.4% similarity) and 43.2% identity (56.3% similarity) to the Och1 protein of *S. cerevisiae* and *C. albicans*, respectively. These values are similar to the homology of the two yeast Och1 proteins (37.3% identity; 51.3% similarity). All three sequences share two stretches of high homology: one around the putative glycosyltransferase sugar-binding region (Pfam domain PF04488), comprising a characteristic DXD motif, and a second in the C-terminal region (Supplementary Figure S1).

### Generation and characterization of a Δ*afoch1* mutant

**Figure 1:**
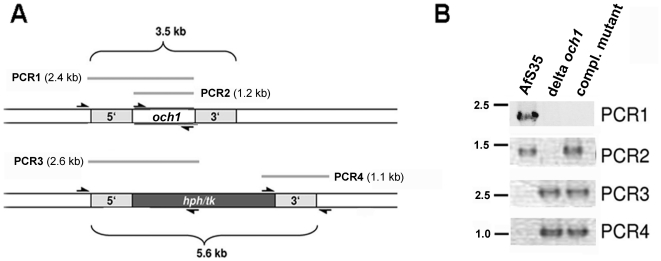
Construction of the Δ*afoch1* mutant and the complemented strain. (A) Structure of the genomic *afoch1* gene and the deleted *afoch1*::*hph/tk* locus. Approximately 1 kb of the 5′ and 3′ regions of *afoch1* (gray boxed areas) were used for construction of the deletion cassette. The positions of the primers used for PCR amplifications and the resulting PCR products (PCR 1–4) are indicated. (B) Equal amounts of genomic DNA of AfS35, Δ*afocht1* and Δ*afoch1* + *afoch1* were used as template for PCR amplification of the regions indicated in panel A (PCR 1–4).

A deletion construct was generated that comprises 1 kb regions up- and down-stream of the *afoch1* gene flanking a hygromycin resistance cassette (Figure 1A). This linear construct was used for transformation of protoplasts of strain AfS35 [10]. PCR analysis of a hygromycin-resistant clone that was used throughout this study is shown in Figure 1B. Single integration of the deletion construct was verified by Southern blot (data not shown). For complementation the mutant was transformed with a construct that comprises the *afoch1* gene (under the control of the *A. nidulans gpdA* promoter) and a pyrithiamine resistance cassette. Several pyrithiamine resistant clones were obtained and analyzed by PCR. The results obtained for the clone used in subsequent studies are shown in Figure 1B.

**Figure 2:**
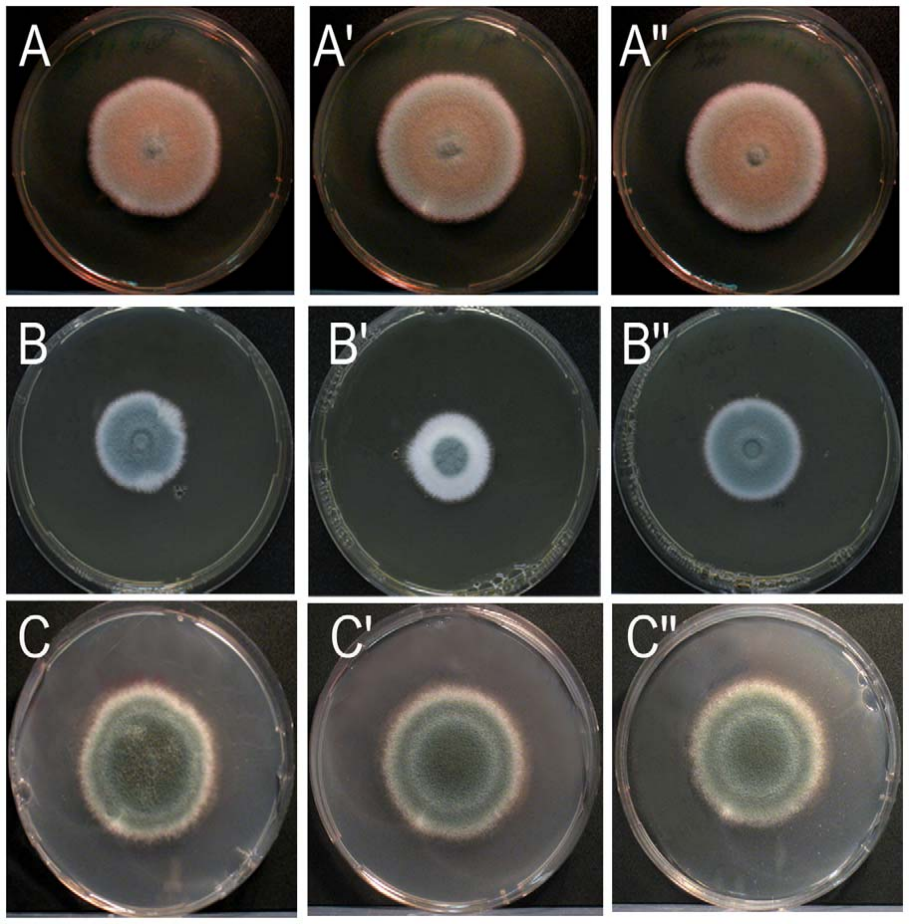
Impaired sporulation of the Δ*afocht1* mutant in the presence of high calcium concentrations. Colonies of the parental strain AfS35 (A, B, C), the Δ*afocht1* mutant (A′, B′, C′) and the complemented strain (A″, B″, C″) were grown at 37°C on YG medium (A to A″), YG medium supplemented with 350 mM CaCl_2_ (B to B″) and AMM medium supplemented with 500 mM CaCl_2_ (C to C″).

**Figure 3:**
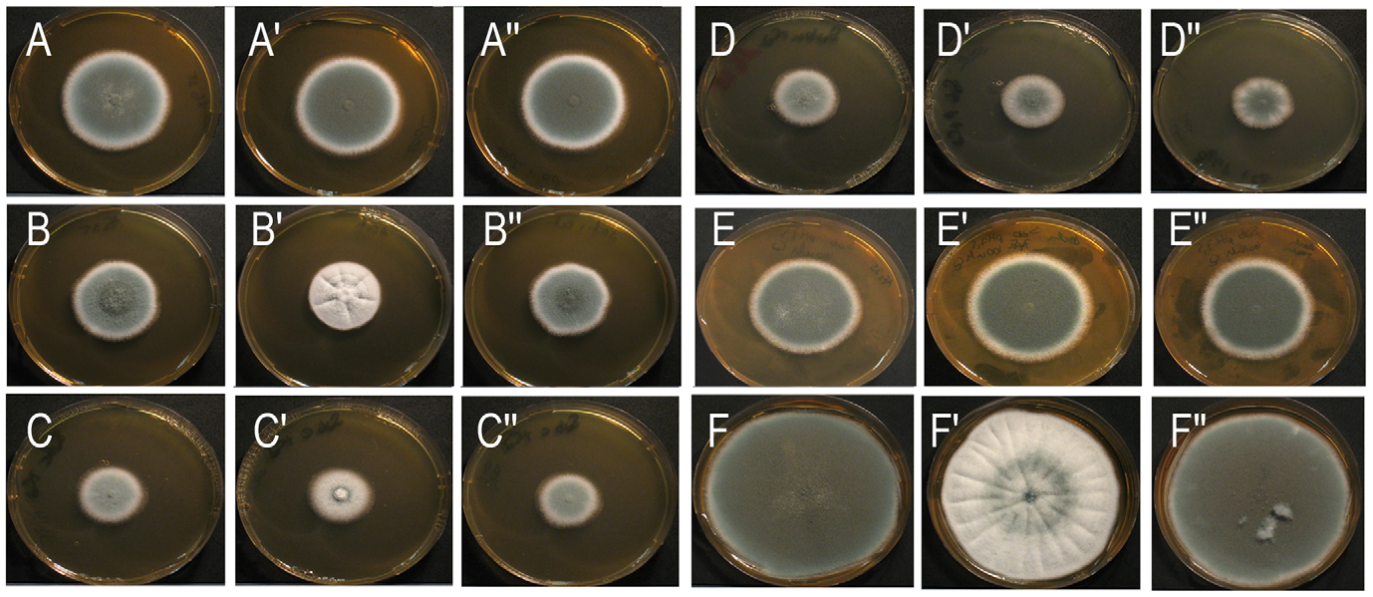
Impaired sporulation of the Δ*afocht1* mutant grown on Sabouraud medium supplemented with calcium. The parental strain AfS35 (A–F), the Δ*afocht1* mutant (A′–F′) and the complemented strain (A″–F″) were cultured on plates with the indicated medium at 37°C for 48 h (A–E) or 240 h (F). A: Sabouraud medium; B and F: Sabouraud medium +100 mM CaCl_2_; C: Casein (10 g/L)+4% Glucose +100 mM CaCl_2_; D: Pepton (10 g/L) )+4% Glucose +100 mM CaCl_2_; E: Sabouraud medium +100 mM CaCl_2_, pH 7.0.

The Δ*afoch1* mutant and the control strains (the parental strain AfS35 and the complemented mutant) showed comparable growth and sporulation on AMM, YG and Sabouraud plates at 37°C (Figure 2A–A″, 3A–A″ and data not shown). The *S. cerevisiae och1* mutant is impaired in growth at elevated temperatures [11] and its *C. albicans* counterpart is moreover sensitive to different cell wall stressors, e.g., calcofluor white and SDS [3]. Growth of the Δ*afoch1* mutant was analyzed under these and additional stress conditions. We observed no phenotype at temperatures up to 48°C, on YG plates containing different stressors (calcofluor white (200 µg/ml), Congo red (100 µg/ml), SDS (0.05%), sodium desoxycholate (0.1%)), in a disk diffusion assay using 30% H_2_O_2_, and in E-tests with voriconazol, caspofungin and amphotericin B (data not shown).

### Impaired sporulation of the Δ*afoch1* mutant under high calcium conditions

We recently described that the *A. fumigatus* mannosyltransferase MitA is essential for synthesis of complex mannose:inositol-phosphorylceramide-derived glycolipids and that the corresponding mutant is impaired in growth in the presence of high calcium concentrations [8]. Under similar conditions (YG agar plates containing 350 mM calcium) the colony size of the Δ*afoch1* mutant was only slightly reduced compared to the control strains (Figure 2B–B″), but the mutant colonies showed a striking broad white rim of non-sporulating mycelium (Figure 2B′). No defect in sporulation or growth was detectable on plates containing 350 mM MgCl_2_ (data not shown), demonstrating that this effect is not due to an increased concentration of divalent or chloride ions in the medium.

Further experiments revealed no sporulation phenotype of the mutant on Aspergillus minimal medium (AMM) plates (containing 1% or 4% glucose) supplemented with up to 500 mM calcium (Figure 2 C–C″ and data not shown). This indicates that calcium *per se* is not sufficient to trigger the sporulation defect. On Sabouraud plates the sporulation phenotype was already evident at 100 mM calcium (Figure 3 B–B″) and became even more apparent after 240 h incubation, when colonies of the control strains showed complete area-wide sporulation (Figure 3 F–F″). Sabouraud medium comprises 4% glucose, pancreatic digest of casein and pepton (5 g/L each). To determine whether the complex components (that are not present in AMM) are required, we analyzed growth on plates with 100 mM calcium, 4% glucose and either 10 g/L casein digest or peptone. The sporulation defect was only apparent on plates containing casein (Figure 3 C–C″), demonstrating a requirement for a so far undefined component present in casein, but not in pepton. A characteristic feature of Sabouraud medium is the acidic pH of 5.6. Adjusting the pH to 7.0 abolished the sporulation phenotype (Figure 3 E–E″). Growth of the mutant on AMM plates adjusted to pH values of 5.0, 6.0 and 7.0 revealed no defect in sporulation, demonstrating that a low pH is required but not sufficient to induce the sporulation phenotype of the Δ*afoch1* mutant (data not shown).

### *Aspergillus fumigatus* Och1 is involved in N-glycan biosynthesis

The Och1 proteins of *S. cerevisiae* and *C. albicans* are α1,6-mannosyltransferases acting on the Man_8_GlcNAc_2_ core of N-glycans. In the *C. albicans* Δ*och1* mutant a reduced N-glycosylation of an N-acetyl-glucosaminidase was described [3]. Using sequence homology we identified the putative orthologous *A. fumigatus* N-acetyl-glucosaminidase (AFUA_8G05020) and performed a similar experiment with protein extracts obtained after growth in AMM broth with glucosamine as the sole carbon source. EndoH digestion reduced the apparent molecular weight indicating the presence of N-glycans, but no difference was observed between the Δ*afoch1* mutant and the control strains (data not shown).

**Figure 4:**
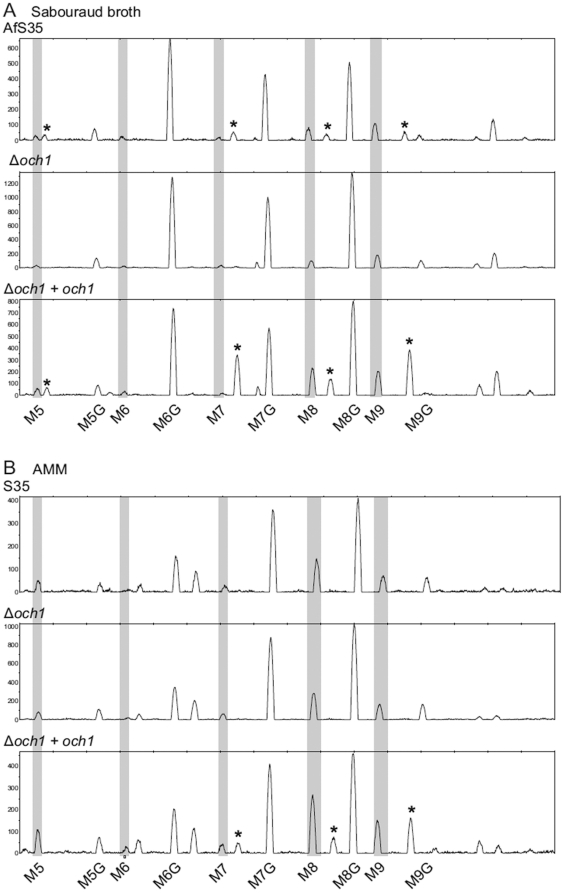
AfOch1 is involved in N-glycan biosynthesis. Electropherograms of fluorescently labelled *N*-glycans enzymatically released from secreted glycoproteins of *A. fumigatus* parental strain AfS35, Δ*afoch1* and Δ*afoch1* + *afoch1* strains grown either in Sabouraud broth (panel A) or Aspergillus minimal media (AMM)(panel B). The *x* axis was calibrated to the fragment sizes of the GeneScan-500 ROX standard (Applied Biosystems). Grey bars indicate the migration of Man_5–9_GlcNAc_2_ core N-glycans used as standards (M5 to M9). Peaks labelled M5G to M9G arise from substitution of these core N-glycans by a single Gal*f* residue as previously described (Schmalhorst *et al*. 2008). Asterisks indicate AfOch1 dependent N-glycans.

Continuing along these lines, N-glycans of secreted proteins from *A. fumigatus* wild type, Δ*afoch1* and Δ*afoch1*+*afoch1* were released by PNGase F treatment and analysed by capillary electrophoresis after fluorescent labelling. As seen in Figure 4 and in previous reports [12], [13], *A. fumigatus* secreted proteins mainly carry N-glycans composed of the Man_5–9_GlcNAc_2_ core predominantly substituted with a galactofuranose residue. Additional N-glycan structures labelled with an asterisk in Figure 4 are observed when *A. fumigatus* is grown in Sabouraud broth (first electropherogram, panel A) but not in minimal media (first spectrum, panel B). Importantly, these peaks are absent from the Δ*afoch1* strain (second electropherogram, panel A) and increased in the complemented mutant (third electropherogram, panel A). In minimal media, these additional N-glycans are only observed if AfOch1 is expressed from the strong *gpdA* promoter of the complementation construct. Moreover, N-glycans of higher molecular weight are also present when *A. fumigatus* is grown in Sabouraud but not in minimal media broth (first and third electropherogram of the Supplementary Figure S2). The majority of these structures (labelled with an asterisk) are dependent on AfOch1expression. These data clearly indicate the involvement of AfOch1 in the biosynthesis of N-glycans. They suggest that synthesis of polymannosylated N-glycans occurs in *A. fumigatus* and might be triggered in response to specific environmental conditions. These results would be in agreement with AfOch1 playing a similar role as *S. cerevisiae* ScOch1 in the initiation of an outer chain.

### Infection experiments

**Figure 5:**
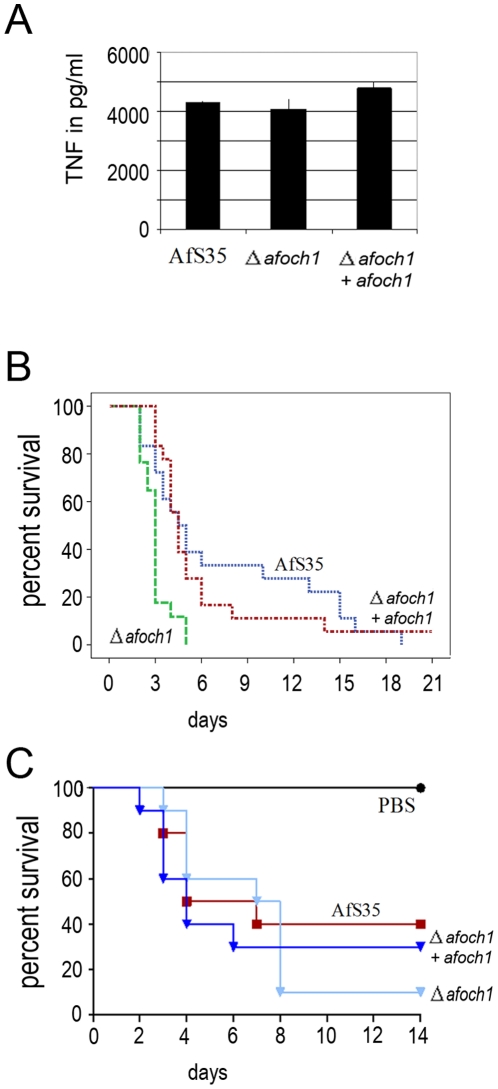
Characterization of the Δ*afocht1* mutant in infection experiments. (A) The TNFα responses of murine bone marrow-derived macrophages infected with the wild type, the Δ*afoch1* mutant and the complemented mutant are shown in panel A. (B) Intranasal infection of cortisone-treated mice infected with 1×10^6^ conidia each of the Δ*afoch1* mutant (n = 10), the parental strain Afs35 (n = 10) and the complemented strain (n = 10). Survival of mice is depicted over time. (C) Systemic infection model using immunocompetent CD-1 mice. Mice were infected retroorbitally with 2×10^6^ conidia of the Δ*afoch1* mutant (n = 17), the parental strain Afs35 (n = 18) and the complemented strain (n = 18). Survival of mice is depicted over time.

The *och1* mutant of *C. albicans* was shown to induce a reduced cytokine response in murine macrophages in comparison to the corresponding wild type strain [4]. Infection of murine, peritoneal macrophages with the Δ*afoch1* and the control strains revealed no significant difference in the levels of secreted TNFα, IL-6 or IL-10 (Figure 5A and data not shown). To analyze whether the Δ*afoch1* mutant is attenuated in virulence, as reported for its *C. albicans* counterpart [3], we infected mice using a systemic model of infection with immunocompetent mice and an intranasal infection model with immunocompromised mice. We observed no attenuation for the Δ*afoch1* mutant in either model of infection (Figure 5B and C). Unexpectedly, infections with the Δ*afoch1* led to a significantly faster killing in the systemic model of infection (p = 0.002, Figure 5B) and indications of a slightly higher virulence were also observed in the intranasal infection model (Figure 5C).

### Och1-like proteins of yeast and filamentous fungi differ in their N-termini

Yeasts and filamentous fungi usually harbor several members of the Och1-family. The Hoc1 proteins are **h**omologous to **Oc**h1 and build a second, phylogentically distinct subfamily of putative mannosyltransferases. ScHoc1 resides in the Golgi, but the gene is unable to complement an *S. cerevisiae* Δ*och1* mutant [14]. Another member of the Och1-family, ScOcr1 (**Oc**h1-**r**elated protein), was shown to contribute to the elongation of N-glycan outer chains and to O-glycosylation of proteins [15]. A phylogenetic tree derived from the protein sequences of Och1, Hoc1 and Ocr1 proteins from *S. cerevisiae*, *P. angusta*, *C. albicans* and *A. fumigatus* is shown in the Supplementary Figure S3. As expected, families of Och1 and Hoc1 proteins are apparent, but apart from AfOch1 none of the Och proteins of *A. fumigatus* can be allocated to one of these subfamilies. In fact, AfOch3 appears to be more closely related to the GDP-mannose:inositol-phosphorylceramide–mannosyltransferase AfMitA than to the other putative or proven α1,6-mannosyltransferases (Supplementary Figure S3).

**Figure 6:**
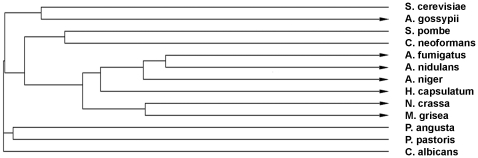
Phylogenetic tree of Och1 proteins. Sequences of Och1 proteins of the indicated fungi were analyzed using ClustalW2 (The corresponding accession numbers are given in Suppl. Table S1). Arrowheads indicate a prediction of an N-terminal signal sequence; blunt ends indicate a prediction for an N-terminal membrane anchor.

A closer inspection of the AfOch1 polypeptide sequence revealed a remarkable difference to its yeast orthologs. All yeast Och1 proteins are predicted to contain N-terminal membrane anchors, whereas a cleavable signal peptide is predicted for AfOch1. We extended this analysis to other ScOch1 homologues. The results are summarized in Figure 6 (more detailed information is given in the Supplementary Table S1). N-terminal membrane anchors are predicted for the Och1-like proteins of *S. cerevisiae*, *C. albicans*, *Schizosaccharomyces pombe*, *Pichia pastoris*, *Pichia angusta* (formerly: *Hansenula polymorpha*) and *Cryptococcus neoformans*, whereas N-terminal signal sequences are predicted for the Och1 proteins of *A. fumigatus*, *A. nidulans*, *A. niger*, *Neurospora crassa*, *Magnaporthe grisea*, *Histoplasma capsulatum* and *Ashbya gossypii*. Strikingly, N-terminal membrane anchors are found in yeast, whereas signal peptides are predicted for filamentous fungi. The Och1 protein of *Ashbya gossypii* is of particular interest. *Ashbya gossypii* belongs to the Saccharomycetaceae and this relationship is also evident from the phylogenetic tree shown in Figure 6. However, *A. gossypii* grows only in the filamentous form and AgOch1 harbors an N-terminal signal sequence. In conclusion, these findings suggest a link between filamentous growth and Och1 proteins having a signal peptide.

### The AfOch4 protein

In the *A. fumigatus* Och family signal peptides are only predicted for AfOch1 and AfOch4 (Supplementary Table S2). This prompted us to analyze the putative mannosyltransferase AfOch4 in more detail. Information on the generation and complementation of a Δ*afoch4* mutant is given in Supplementary Figure S4. The mutant grows normally and showed no phenotype under all conditions tested for the *afoch1* mutant including high calcium concentrations (data not shown). Transformation of the Δ*afoch1* mutant with the *afoch4* gene (under the control of the constitutive *gpdA* promoter) did not rescue the sporulation phenotype on calcium plates (Supplementary Figure S4, panel C) suggesting that AfOch1 and AfOch4 have different biological functions.

### Localization of the AfOch1 protein

**Figure 7:**
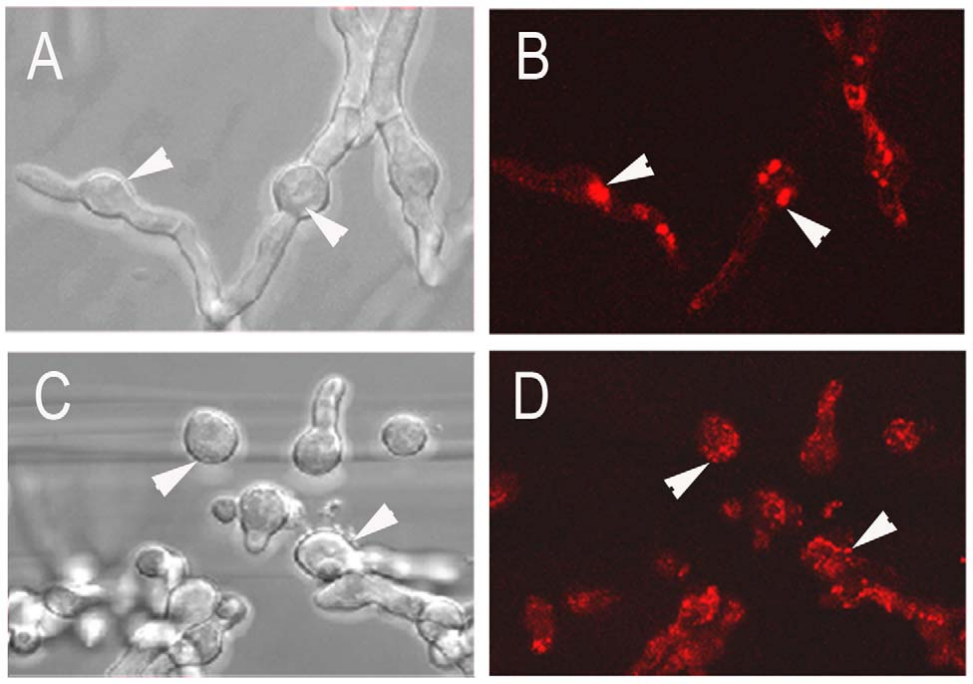
Localization of an AfOch1-RFP fusion protein. Fluorescence microscopy of hyphae expressing an AfOch1-RFP fusion protein (B and D) and overlays with the corresponding bright field images (A and C). The cultures shown in C and D were treated with Brefeldin A, panels A and B show the untreated control.

The ScOch1 protein comprises an N-terminal membrane anchor and is localized in the *cis*-Golgi (Nakayama *et al*., 1992). After cleavage of its N-terminal signal peptide, AfOch1 is released into the lumen of the ER and can thereby enter the secretory pathway. To investigate its localization we fused the *afoch1* gene in frame to the 5′-end of the *rfp* gene (encoding the red fluorescent protein). This construct was able to rescue the sporulation defect of the Δ*afoch1* mutant (data not shown). Microscopic analysis revealed a strong RFP fluorescence that was focused in distinct intracellular compartments (Figure 7A and B). These large spot-like structures disappeared after treatment with Brefeldin A (Figure 7C and D), which is known to disrupt the Golgi apparatus [16]. This demonstrates that AfOch1 is retained in the Golgi although it lacks an N-terminal membrane anchor.

### The functional importance of the AfOch1 N-terminus

**Figure 8:**
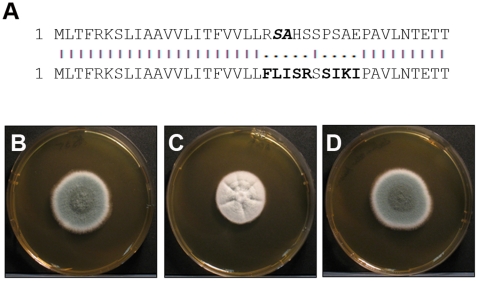
Complementation of the Δ*afocht1* mutant with the *afoch*1* gene lacking a signal sequence. Panel A: Alignment of the forty N-terminal amino acids of AfOch1 and AfOch1*. Amino acids that are mutated in the AfOch1* sequence are indicated in bold. The predicted cleavage site of the signal sequence of AfOch1 is indicated in bold italics (***SA***). Panels B to D: The images show colonies grown on Sabouraud medium supplemented with 100 mM CaCl_2_. The following strains were analyzed: the parental strain AfS35 (A), the Δ*afocht1* mutant (B) and the Δ*afocht1* mutant complemented with the *afoch*1* gene (C). Panel D demonstrates the successful restoration of wild type sporulation in the presence of high calcium concentrations.

To determine whether the signal peptide of AfOch1 is required for it to function properly, we exchanged nine amino acids flanking the predicted cleavage site by mutagenesis of the complementation plasmid (Figure 8A). The resulting AfOch1* is predicted to contain a non-cleavable N-terminal anchor sequence (Signal peptide probability: 0.117; Signal anchor probability: 0.883). Transformation of the Δ*afoch1* mutant with the afoch1* gene rescued the sporulation defect on calcium plates (Figure 8 B–D), indicating that the N-terminal signal sequence is not essential for the function of AfOch1.

## Discussion

The N-glycosylation of eukaryotic proteins proceeds in several steps: synthesis of the core glycan on the lipid carrier dolichylpyrophosphate, its transfer to the nascent protein chain and finally the elongation and trimming of the protein-associated glycan in the Golgi [17]. Differences in protein N-glycosylation between fungi and man are well known and hamper production of recombinant human proteins in filamentous fungi [18]. On the other hand, this difference is exploited by the human immune system to track down fungal pathogens [4]. N-glycans in humans comprise a wide range of structures containing diverse sugars such as fucose, sialic acid and galactose, whereas fungal N-glycans are mainly composed of mannosyl residues. In yeast branching of the outer chain is initiated by the α1,6-mannosyltransferase Och1 and gives rise to characteristic high-mannose structures [1]. The genes of the N-glycan synthesis pathway were identified and characterized in yeast and seem to be well conserved in filamentous fungi [6].

In this study we have characterized a mutant in the Och1 orthologue of *A. fumigatus*. Deletion of the *afoch1* gene had no obvious consequence for growth under standard or diverse stress conditions, e.g., cell wall, oxidative, osmotic and thermal stress. A recent study also failed to disclose a phenotype for a Δ*afoch1* mutant, although transcription of *afoch1* was proven by RT-PCR [19]. We also detected *afoch1* transcription during hyphal growth. Sequencing of the corresponding cDNA revealed an error in the annotation of *afoch1* in the genome sequence that was also noticed by Lambou *et al*. [19]. By testing various growth conditions, we finally identified a phenotype for the Δ*afoch1* mutant. As it turned out, the AfOch1 protein is required for normal sporulation in the presence of high concentrations of calcium. This phenotype was evident on Sabouraud and YG plates, although at different calcium concentrations. On Sabouraud plates the sporulation defect was already apparent at 100 mM calcium, whereas a concentration of 350 mM calcium was required on YG plates. Closer microscopic inspection of the colonies revealed a drastically reduced number of conidiophores in the white areas of the Δ*afoch1* colonies. No sporulation defect was observed on minimal medium (AMM), suggesting an impact of the complex components of Sabouraud and YG. Sabouraud broth comprises glucose, peptone and casein digest and is characterized by a pH of 5.6. The sporulation phenotype appears to be dependent on the elevated calcium concentration, a so far undefined component present in casein and a low pH. Each element *per se* does not appear to be sufficient to cause the phenotype. Calcium could not be substituted by magnesium, suggesting a specific requirement for this ion. Recently, Zanni and co-workers described a link between Och1 and Ca^2+^/calmodulin-based signaling in *Kluyveromyces lactis* [20]. This interaction is required to maintain proper mitochondrial functionality. Since growth of *A. fumigatus* is strictly dependent on respiration, a reduced mitochondrial functionality should have consequences for fungal growth. This is obviously not the case, indicating a substantial difference between *A. fumigatus* and *K. lactis.* However, it is remarkable that in both fungal organisms Och1 function seems to be somehow linked to calcium homeostasis.

Although genes of the N-glycan pathway are conserved in filamentous fungi, there hasn't been any experimental evidence yet demonstrating complex, poly-mannosylated N-glycans in filamentous fungi. So far, the limited number of published N-glycan structures of *Aspergillus* provided no convincing evidence for the existence of elaborated outer chains [21]–[25]. In this study we demonstrate that poly-mannosylated N-glycans are formed by *A. fumigatus* under certain circumstances. Evidence for outer chains was obtained for the wild type strain, but not for the mutant, indicating that AfOch1 is essentially required. Evidence for poly-mannosylated N-glycans was detectable for the wild type after growth in Sabouraud, but not in AMM broth, whereas the complemented strain synthesized these glycostructures in both media. Expression of *och*1 in the complementation construct is driven by the constitutive *gpd*A promoter and is therefore not completely restoring the wild type situation. However, this constitutive expression led to the formation of outer chains in AMM broth and thereby provided a valuable hind that expression of *afoch1* from its native promoter is strongly influenced by the growth medium. AfOch4 a homolog of AfOch1 is not able to complement the Δ*afoch1* mutant. Whether AfOch2 or AfOch3 play a role in the initiation or elongation of N-glycan outer chains remains to be determined.

A *C. albicans och1* mutant is impaired in virulence and triggers lower levels of proinflammatory cytokines during infection of murine macrophages [3], [4]. Similar infection experiments revealed no difference between the Δ*afoch1* mutant and the control strains, suggesting that N-glycan outer chains of *A. fumigatus* are no relevant target for murine pattern recognition receptors. Our findings may reflect a fundamental difference between the cell wall of yeast and *A. fumigatus*. A thick layer of mannosylated proteins on the core cell wall is characteristic for the former, but seems to be absent in *Aspergillus*. Since *afoch*1 expression is dependent on the growth conditions, AfOch1 may not be expressed during *in vitro* infection of macrophages. However, these experiments do not necessarily reflect the situation in the infected host. Analysis of the virulence of the Δ*afoch1* mutant in two murine models of infection revealed no attenuation. Surprisingly, infection with the mutant led to a faster killing in the systemic model of infection. Whether this truly reflects a slight increase in virulence remains to be determined. For the time being our data at least suggest that AfOch1 is expressed during infection. To prove this, specific antibodies for the outer chains of *A. fumigatus* N-glycans would be valuable tools.

In contrast to the Och1 protein of *S. cerevisiae* AfOch1 harbors a signal sequence that is most likely cleaved off during transport over the ER membrane. This difference prompted us to analyze other members of the Och1-family. Strikingly, the Och1 proteins of the filamentous fungi *A. fumigatus*, *A. nidulans*, *A. niger*, *N. crassa* and *M. grisea* harbor signal sequences, whereas the Och1 protein from the yeasts *S. cerevisiae*, *S. pombe*, *P. angusta*, *P. pastoris* and *C. albicans* have N-terminal anchor sequences. *Ashybia gossypii* is closely related evolutionarily to *S. cerevisiae,* and both Och1 proteins are highly homologous, but AgOch1 harbors a signal peptide. Interestingly, *A. gossypii* grows in hyphal filaments and has been described as “filamentous yeast” [26]. *Candida albicans, S. cerevisiae* and *H. capsulata* can switch between a hyphal and a yeast form. The two former have anchor sequences, while the latter is strongly predicted to harbor a signal peptide. *Histoplasma capsulata* commonly grows in filamentous hyphae, but switches to a pathogenic yeast morphotype at 37°C. Hence, it is tempting to speculate that the presence of an N-terminal signal peptide is somehow linked to a hyphal morphology under conventional growth conditions. It is interesting to note that the elaborated outer chains of *S. cerevisiae* and *C. albicans* were all isolated from the yeast morphotype [3], [11]. Whether outer chains are abundant in the pathogenic hyphal morphotype of *C. albicans* remains to be determined.

After cleavage of its N-terminal signal sequence, AfOch1 is released into the lumen of the ER and may travel to the cell surface. However, enzymes that are required for N-glycan synthesis conventionally reside in the Golgi. They are generally supposed to be membrane-anchored and to interact with luminal target proteins [27]. We have localized an RFP-fusion of AfOch1 in distinct intracellular compartments. The sensitivity of these structures to Brefeldin A strongly suggests that they represent the *Aspergillus* Golgi apparatus. Proper localization of the fusion protein was verified by its ability to complement the sporulation defect of the Δ*afoch1* mutant. In yeast, the localization of ScOch1 in the Golgi involves retrograde transport of this membrane anchored protein [28]. However, similar mechanisms operate with other Golgi mannosyltransferases, e.g., ScHoc1, which harbors a signal peptide [14]. Thus, so far unknown proteins may assist ScHoc1 and AfOch1 to remain at the membrane of the Golgi. A mutated AfOch1* protein that lacks the cleavage site and is predicted to harbour an N-terminal membrane anchor instead is able to rescue the sporulation defects of the Δ*afoch1* mutant. These data indicate redundant sorting mechanisms that assure that Golgi proteins remain in this organelle, which is also suggested by the finding that a mutated form of ScMnn1 lacking its N-terminal membrane anchor is retained in the Golgi [29]. It is possible that the conserved stretch of amino acids in the C-terminal part of AfOch1 is involved in protein-protein interactions that mediate this sorting mechanism.

In conclusion, we provide evidence that AfOch1 is expressed in *A. fumigatus* and that this expression depends on the growth medium. Under permissive conditions outer chains are detectable in the N-glycans of *A. fumigatus.* This strongly suggests that AfOch1, like its yeast counterparts, is an α1,6-mannosyltransferase that initiates the branching of the outer chain. A mutant lacking this enzyme showed no phenotype in plate assays under standard and stress conditions. However, it is strongly impaired in sporulation in the presence of high calcium concentrations. The precise mechanism underlying this phenotype is unknown, but it provides a convenient mean to analyze AfOch1 activity. In AfOch1 a signal sequence replaces the N-terminal membrane anchor that is characteristic for Och1 proteins in yeast. This seems to be generally the case in filamentous fungi, suggesting a link between the growth in filamentous hyphae and the presence of an N-terminal signal sequence in Och1 proteins. Why Och1 proteins differ in their N-termini and how this correlates to a different abundance of elaborated outer chains in yeast and filamentous fungi is still an open question, but our results provide a base to analyze this in more detail.

## Materials and Methods

### Strains and culture conditions

The *A. fumigatus* Δ*aku*A strain AfS35, the procedure for isolation of conidia, and the composition of the yeast glucose medium (YG) and the Aspergillus Minimal Medium (AMM) have been described previously [10], . Sabouraud medium, casein and pepton were purchased from Becton-Dickinson.

### Sequence analysis and data base searches

Database searches were performed using BlastP and BlastN and the following databases: GeneBank/EMBL/DDBJ, the Central Aspergillus Data Repository (CADRE) (http://www.cadre-genomes.org.uk/Aspergillus_fumigatus/), the Saccharomyces Genome Database (http://www.yeastgenome.org/) and the Candida Genome Database (http://www.candidagenome.org/). Predictions of signal sequences were performed using SignalP 3.0 (http://www.cbs.dtu.dk/services/SignalP/). N-glycosylation sites were predicted using NetNGlyc 1.0 (http://www.cbs.dtu.dk/services/NetNGlyc/). Predictions for O-β-GlcNAc attachment sites were performed using YinOYang (http://www.cbs.dtu.dk/services/YinOYang/). Sequence homologies were analyzed using the EMBOSS Pairwise Alignment Algorithms (http://www.ebi.ac.uk/Tools/emboss/align/). Prediction of protein domains was performed using InterProScan Sequence Search (http://www.ebi.ac.uk/Tools/InterProScan/). Phylogenetic trees were obtained using CLUSTAL (http://www.ebi.ac.uk/Tools/clustalw2/).

### Genetic nomenclature

At a meeting in Copenhagen in 2004 the *Aspergillus fumigatus* genome sequencing group proposed a nomenclature based on the Demerec bacterial system that should be used for all species (i.e. *abd*D for genes). In our manuscript we followed their recommendations.

### Construction of the Δ*afoch1* and Δ*afoch4* mutants and the complemented strains

All oligonucleotides used in this study are summarized in Supplementary Table S3. The *afoch1*/AFUA_5G08580 gene was amplified from cDNA using oligonucleotides och1-5′ and och1-3′. The PCR product was cloned, sequenced and submitted to the data base.

To construct a suitable replacement cassette a 3.5 kb hygromycin resistance cassette was excised from pSK346 using the SfiI restriction enzyme. The flanking regions of the *afoch1* gene (approx. 1.0 kb each) were amplified by PCR from chromosomal DNA using the oligonucleotide pairs och1-upstream-5′/och1-upstream-3′ and och1-downstream-5′/och1-downstream-3′. These oligonucleotides harbored incompatible SfiI sites. After digestion with SfiI a ligation of the three fragments (resistance cassette and flanking regions) yielded a 5.4 kb deletion cassette that was purified using the PrepEase Gel Extraction Kit (USB, Cleveland, USA). The fragment was cloned into the pBluescriptKS vector (Stratagene, La Jolla, USA) using oligonucleotide derived NotI sites. Purified NotI fragments from the resulting plasmid were used for transformation.

For complementation the *afoch1* gene was amplified using oligonucleotides och1-5′ and och1-3′. The PCR product was purified and cloned into pSK379 to drive expression from the *gpdA* promoter. The resulting plasmid was isolated using the Pure Yield Plasmid Midiprep System (Promega, Mannheim, Germany). *A. fumigatus* protoplasts were generated and transformation was performed essentially as described previously [9]. The resulting protoplasts were transferred to AMM plates containing 1.2 M sorbitol and either 200 µg/ml hygromycin (Roche, Applied Science, Mannheim, Germany) or 0.1 µg/ml pyrithiamine (Sigma-Aldrich, Deisenhofen, Germany).

The generation of the Δ*afoch4* mutant was performed accordingly using oligonucleotides summarized in Supplementary Table S3.

### Genomic DNA analysis

*A. fumigatus* clones which showed the expected resistance on selective plates were further analyzed by PCR. In the first PCR, one oligonucleotide (och1-cast-5′) that hybridized immediately upstream of the *afoch1* gene was combined with a second primer (och1-3′) localized at the 3′ end of the *afoch1* gene (compare Fig. 1). This reaction (PCR1) was used to detect the *afoch1* gene in its wild type context. The core *afoch*1 gene was amplified using oligonucleotides och1-5′ and och1-3′ (PCR2). The correct integration of the deletion cassette was analyzed at the 5′ end using oligonucleotides och1-cast-5′ and hph-3-SmaI (PCR3) and at the 3′ end using oligonucleotides trpCt-fwd and och1-3′UTR-rev (PCR4) (Figure 1).

Mutation of the 5′-end of the *afoch1* gene corresponding to the cleavage site of the signal peptide was performed using the above described complementation plasmid. An inverse long range PCR using the oligonucleotides och1-TMD-5′ and och1-TMD-3′ and the Long Amp *Taq* Polymerase (New England Biolabs) was performed according to the instructions of the vendor. The resulting 9554 bp fragment was subsequently purified and blunted using T4 DNA Polymerase (Fermentas). After phosphorylation the fragment was ligated given rise to a mutated complementation vector. After sequencing this vector was introduced into the Δ*afoch*1 mutant.

### Phenotypic testing on plates

Isolated conidia were counted using a Neubauer chamber. For drop dilution assays, series of tenfold dilutions derived from a starting solution of 1×10^8^ conidia per ml were spotted in aliquots of 3 µl onto plates. These plates were supplemented with the indicated agents and incubated at the indicated temperatures. For quantification of the radial growth, 3 µl containing 1×10^6^ conidia were spotted in the center of a 9 cm Petri dish. The diameter of the colonies was determined over time.

E-test strips of voriconazole, amphotericin B and caspofungin were obtained from Inverness Medical (Cologne, Germany) and used as described previously [9].

### N-glycan analysis

N-glycan preparation and separation was carried out essentially as described previously [31]. Briefly, glycoproteins from 1 mL supernatant of *A. fumigatus* cultures grown for 3 days at 37°C were transferred to Immobilon P Multiwell plates (Millipore). After N-glycan release with peptide:N-glycanase (PNGase F, New England Biolabs) in 50 mM NH_4_HCO_3_ pH 8.4 and labelling with 8-amino-1,3,6-pyrenetrisulfonic acid, N-glycans were separated on a capillary electrophoresis DNA Sequencer (ABI PRISM® 3100-Avant Genetic Analyzer, Applied Biosystems, Foster City, CA, USA) using an injection time of 30 to 90 s. Reference glycans were purchased from Dextra Laboratories (Reading, UK).

### Construction and analysis of strains expressing an AfOch1-RFP fusion proteins

The *afoch1* gene from nucleotide 1 to 1053 was amplified using oligonucleotides och1-5′ and och1-3′ b. The resulting 1053 bp fragment was cloned into pJW101 using the PmeI restriction site. This derivative of pSK379 comprises the gene for the monomeric red fluorescence protein 1 (mRFP1) [9]. The resulting plasmid pJW102 was transformed into *A. fumigatus* protoplasts as described above. To test whether the AfOch1-RFP-containing organelles are sensitive to Brefeldin A, hyphae grown in minimal medium (AMM) at 37°C were incubated for 4 h in the presence of 20 µg/ml BrefeldinA (Sigma, Deisenhofen, Germany). Microscopic analysis analysis was performed using a SP-5 confocal laser scanning microscope (Leica Microsystems, Heidelberg, Germany).

### Infection of isolated murine macrophages

Primary resting murine macrophages were isolated by peritoneal lavage of C57/Bl6 mice using standard procedures. The subsequent cultivation was performed using RPMI1640 medium supplemented with 5% fetal calf serum. Cells were seeded in 96 well plates at a density of 2.5×10^4^ cells per well. On the next day, the cultures were infected with 2.5×10^5^ conidia per well. After 15 h, the supernatants were harvested and concentrations of TNFα, IL-10 and IL-6 were determined using the Cytometric Bead Array (Becton Dickinson, Heidelberg, Germany) according to the instructions of the vendor.

### Mouse infection experiments

All animal experiments were performed in accordance to the national regulations in Germany. Analysis of the mutant in a systemic model of infection was essentially performed as described previously (Wagener *et al*., 2008). Briefly, 2×10^6^ conidia of the Δ*afoch1* mutant, the complemented mutant or the parental strain AfS35 in a final volume of 300 µl PBS containing 0.02% Tween 20 were injected retroorbitally into male CD-1 mice. Survival of infected animals was monitored once a day.

Alternatively, we used an intranasal infection model with immunocompromized female outbred CD-1 mice. Briefly, mice were immunosuppressed by intraperitoneal injection of cortisone acetate (25 mg/mouse, Sigma-Aldrich) on days −3 and 0. On day 0 the mice were anesthetized with fentanyl (0.06 mg/kg, Janssen-Cilag, Germany), midazolam (1.2 mg/kg, Roche, Germany) and medetomidin (0.5 mg/kg, Pfizer, Germany) and infected intranasally with 1×10^6^ conidia in 20 µl PBS. Controls received PBS only. Survival was monitored for 14 days. During this period, mice were examined clinically at least twice daily and weighed individually every day.

Kaplan-Meier survival curves were compared using the log rank test (SPSS 15.0 software). P values <0.05 were considered statistically significant.

## Supporting Information

Figure S1 **Sequence alignment of the Och1 proteins of** ***Saccharomyces cerevisiae*** **(ScOch1; YGL038C),** ***Candida albicans*** **(CaOch1; orf19.7391) and** ***A. fumigatus*** **(AfOch1; AFUA_5G08580).** The Pfam domain PF04488 is indicated in red and the DXD motif is underlined.
(DOC)

Figure S2 **The presence of larger N-glycans in** ***A. fumigatus*** **requires Och1 expression and depends on culture conditions.** Electropherograms of fluorescently labelled N-glycans enzymatically released from secreted glycoproteins of *A. fumigatus* parental strain AfS35, Δ*afoch1* and Δ*afoch1* + *afoch1* strains grown either in Sabouraud broth or Aspergillus minimal media. The *x* axis was calibrated to the fragment sizes of the GeneScan-500 ROX standard (Applied Biosystems). The grey bar indicates the migration of the Man_9_GlcNAc_2_ core N-glycan used as standard (M9). Asterisks indicate AfOch1 dependent N-glycans.
(TIF)

Figure S3 **Probability of N-terminal signal sequences and membrane anchors of selected proteins of the Och1 family from** ***A. fumigatus*** **,** ***S. cerevisiae*** **,** ***C. albicans*** **and** ***P. angusta.*** The sequences were analyzed using the SignalP 3.0 algorithm. The accession numbers of probabilities for a signal sequence or a membrane anchor is given in Panel A. A phylogenetic tree derived from these sequences using ClustalW2 is shown in Panel B. Arrowheads indicate the prediction of a signal peptide, blunt ends indicate a predicted N-terminal membrane anchor. Uncertain predictions are indicated by a question mark.
(TIF)

Figure S4 **Construction of the Δ** ***afoch4*** **mutant and its complementation.** (A) Structure of the genomic *afoch4* gene and the deleted *afoch1*::*hph/tk* locus. Approximately 1 kb of the 5′ and 3′ regions of *afoch4* (gray boxed areas) were used for construction of the deletion cassette. The positions of the primers used for PCR amplifications and the resulting PCR products (PCR 1–4) are indicated. (B) Equal amounts of genomic DNA of AfS35, Δ*afocht4* and Δ*afoch4* + *afoch4* were used as template for PCR amplification of the regions indicated in panel A (PCR 1–4). (C) The *afoch4* gene is unable to restore wild type sporulation in a Δ*afocht1* mutant. Colonies grown on Sabouraud medium +100 mM CaCl_2_ at 37°C are shown for the parental strain AfS35 (C), the Δ*afoch1* mutant (C′) and the Δ*afoch1* + *afoch4* strain.
(TIF)

Table S1 **Probability of N-terminal signal sequences and membrane anchors of Och1 proteins.** The Och1 sequences of the indicated fungal species were analyzed using the SignalP 3.0 algorithm. The accession numbers of the analyzed sequences and their homology to the reference ScOch1 protein sequence are given.
(DOC)

Table S2 **Probability of N-terminal signal sequences and membrane anchors for members of the Och1 family of** ***A. fumigatus*** **,** ***S. cerevisiae*** **,** ***C. albicans*** **, and** ***P. augusta*** **.** The Och1 sequences of the indicated fungal species were analyzed using the SignalP 3.0 algorithm. The probabilities of N-terminal signal peptides and membrane anchors, as well as the homologies to the reference ScOch1 protein sequence are given.
(DOC)

Table S3 **Oligonucleotides used in this study.**
(DOC)
