# Supplementary material for: Approaching the Secrets of N-Glycosylation in Aspergillus fumigatus: Characterization of the AfOch1 Protein
Source: PLoS One. 2010 Dec 29;5(12):e15729. doi: 10.1371/journal.pone.0015729 (PMC3012087; doi:10.1371/journal.pone.0015729)
Supplement: Figure S1 — Sequence alignment of the Och1 proteins of Saccharomyces cerevisiae (ScOch1; YGL038C), Candida albicans (CaOch1; orf19.7391) and A. fumigatus (AfOch1; AFUA_5G08580). The Pfam domain PF04488 is indicated in red and the DXD motif is underlined. (DOC) [file pone.0015729.s001.doc]

ScOch1 MSRKLSHLIATRKSKTIVVTVLLIYS---LLTFHLSNKRLLSQFYPSKDDFKQTLLPTTS 57

CaOch1 MLQLREPQMVHKHLKLAVLGIVVIFTTYFIISSLSSPTSTHKTEYNSPKLQLAKELELNS 60

AfOch1 MLTFRKSLIAAVVLITFVVLLRSAHS------SPSAEPAVLNTETTAHDTSQAADEHLTD 54

* . :. *: : .: : . : . ..

ScOch1 HSQDINLKKQITVNKKKNQLHNLRDQLSFAFPYDSQAPIPQRVWQTWKVGADDKNFPSSF 117

CaOch1 NWKELGLNFQPNKKYSLPDESTLRQQLSYQFPYDESKPFPKNIWQTWKVGIDEKSFPKRY 120

AfOch1 QKHDIQQQP-----LKPPPTAPLRERLRYQFPYDLENRFPAYIWQTWKYTPASMWFSEDL 109

: ::: : . **::* : **** . :* :***** . *..

ScOch1 RTYQKTWSGSYSPDYQYSLISDDSIIPFLENLYAPVPIVIQAFKLMPGNILKADFLRYLL 177

CaOch1 LKYQQTWE-DKNPDYKHYVVPDKQCDLLIEQLYSQVPDVAKAYRIMPKSILKADFFRYLI 179

AfOch1 RPAEASWT-ELHPGFVHEVIPDDTQRHLVKYLYGSVPEVFEAYDSMPLPVLKADFFRYLI 168

: :* . *.: : ::.*. ::: **. ** * :*: ** :*****:***:

ScOch1 LFARGGIYS**DMD**TMLLKPIDSWPSQNKSWLNNIIDLNKPIPYKNSKPSLLSSDEISHQPG 237

CaOch1 LFARGGVYT**DID**TVGLKPVDEWIS------NSEMILEK----KN-------------RSG 216

AfOch1 LLARGGIYS**DID**TYALKPAVDWLP-------GELDLAT--------------------VG 201

*:****:*:*:** *** .* . . : * . *

ScOch1 LVIGIEADPDRDDWSEWYARRIQFCQWTIQAKPGHPILRELILNITATTLASVQNPGVPV 297

CaOch1 LVVGIEADPDRPDWADWYARRIQFCQWTIQSKRGHPMLRELIAKITDITLT--------- 267

AfOch1 FVIGIEADPDRPDWHDWYSRRIQFCQWTIQAKPGHPILRDIVAYITEEALR--------- 252

:*:******** ** :**:***********:* ***:**::: ** :*

ScOch1 SEMIDPRFEEDYNVNYRHKRRHDETYKHSELKNNKNVDGSDIMNWTGPGIFSDIIFEYMN 357

CaOch1 ----------------RHKK---GQLKKVLGKN----EGGDIMNWTGPGIFTDTVFEYMN 304

AfOch1 ----------------MKKK---GILKEGKMDK-------TIVEFTGPAAWTDAVFRYFN 286

:*: *. .: *:::***. ::* :*.*:*

ScOch1 NVLRYNSDILLINPNLNKNDEEGSESATTPAKDVDNDTLSKSTRKFYKKISESLQSSNSM 417

CaOch1 NILQS--------PEVFKNKK---------------------------------KWATII 323

AfOch1 N------------PEYFSIEP---------------------------------GSTHNV 301

* *: . . : :

ScOch1 PWEFFSFLKEPVIVDDVMVLPITSFSPDVGQMGAQSSDDKMAFVKHMFSGSWKEDADKNA 477

CaOch1 DWKLFTGMEQPIAIDDVLVLPITSFSPDVNQMGAKDSHDPMAYAKHMFSGSWKDDGMPEM 383

AfOch1 TYEDFTNQQGYKKVGDVVVLPITSFSPGVGQMGAGDLDDPMAFVKHDFSGSWKTDPAL-- 359

:: *: : :.**:*********.*.**** . .* **:.** ****** *

ScOch1 GHK 480

CaOch1 EQ- 385

AfOch1 ---

**Supplementary Figure S1:**

Sequence alignment of the Och1 proteins of *Saccharomyces cerevisiae* (ScOch1; YGL038C), *Candida albicans* (CaOch1; orf19.7391) and *Aspergillus fumigatus* (AfOch1; AFUA_5G08580). The Pfam domain PF04488 is indicated in red and the DXD motif is underlined.
