# Supplementary figures and images for: Approaching the Secrets of N-Glycosylation in Aspergillus fumigatus: Characterization of the AfOch1 Protein
Source: PLoS One. 2010 Dec 29;5(12):e15729. doi: 10.1371/journal.pone.0015729 (PMC3012087; doi:10.1371/journal.pone.0015729)

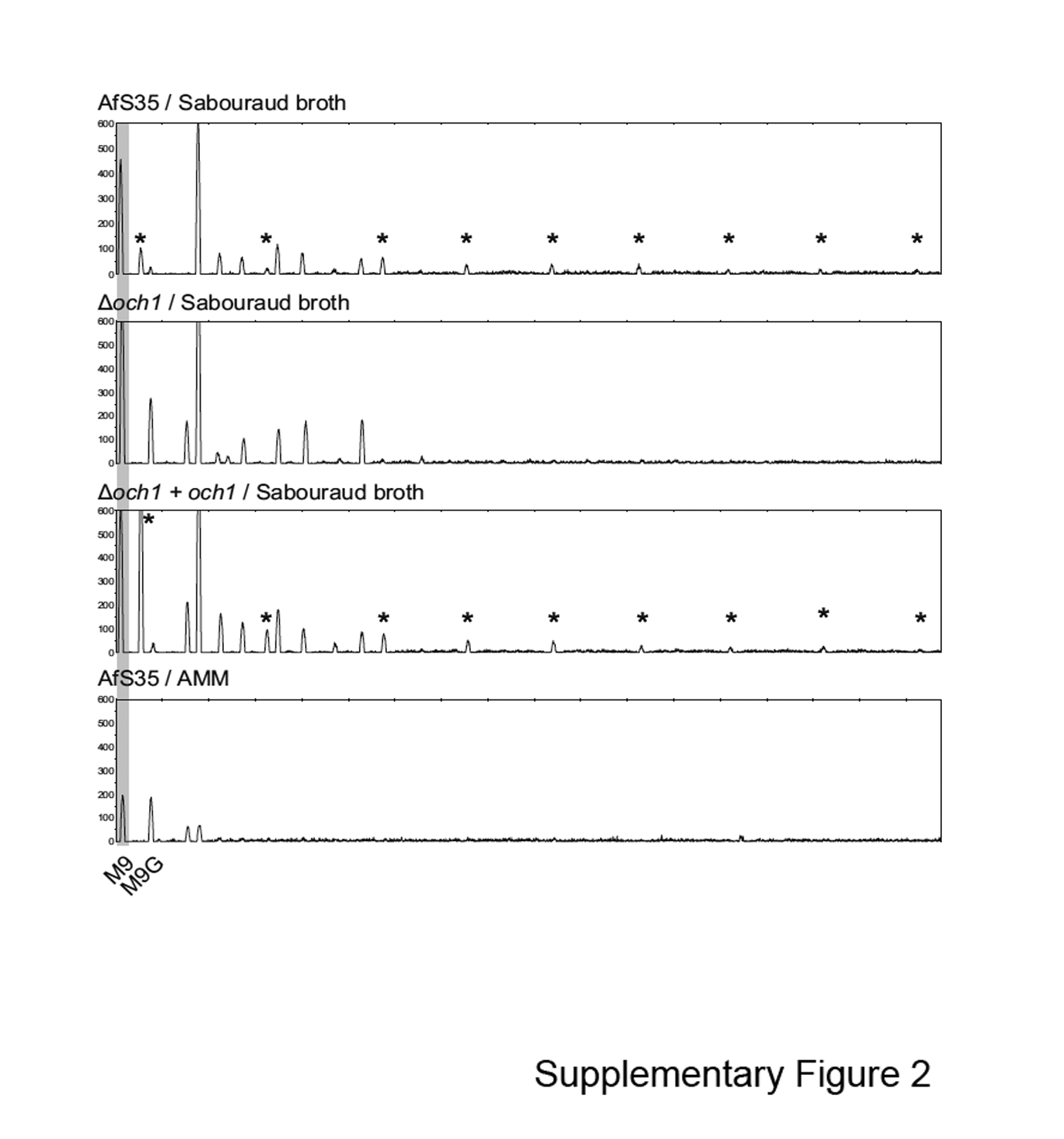

Supplement: Figure S2 — The presence of larger N-glycans in A. fumigatus requires Och1 expression and depends on culture conditions. Electropherograms of fluorescently labelled N-glycans enzymatically released from secreted glycoproteins of A. fumigatus parental strain AfS35, Δafoch1 and Δafoch1 + afoch1 strains grown either in Sabouraud broth or Aspergillus minimal media. The x axis was calibrated to the fragment sizes of the GeneScan-500 ROX standard (Applied Biosystems). The grey bar indicates the migration of the Man9GlcNAc2 core N-glycan used as standard (M9). Asterisks indicate AfOch1 dependent N-glycans. (TIF) [file pone.0015729.s002.tif]

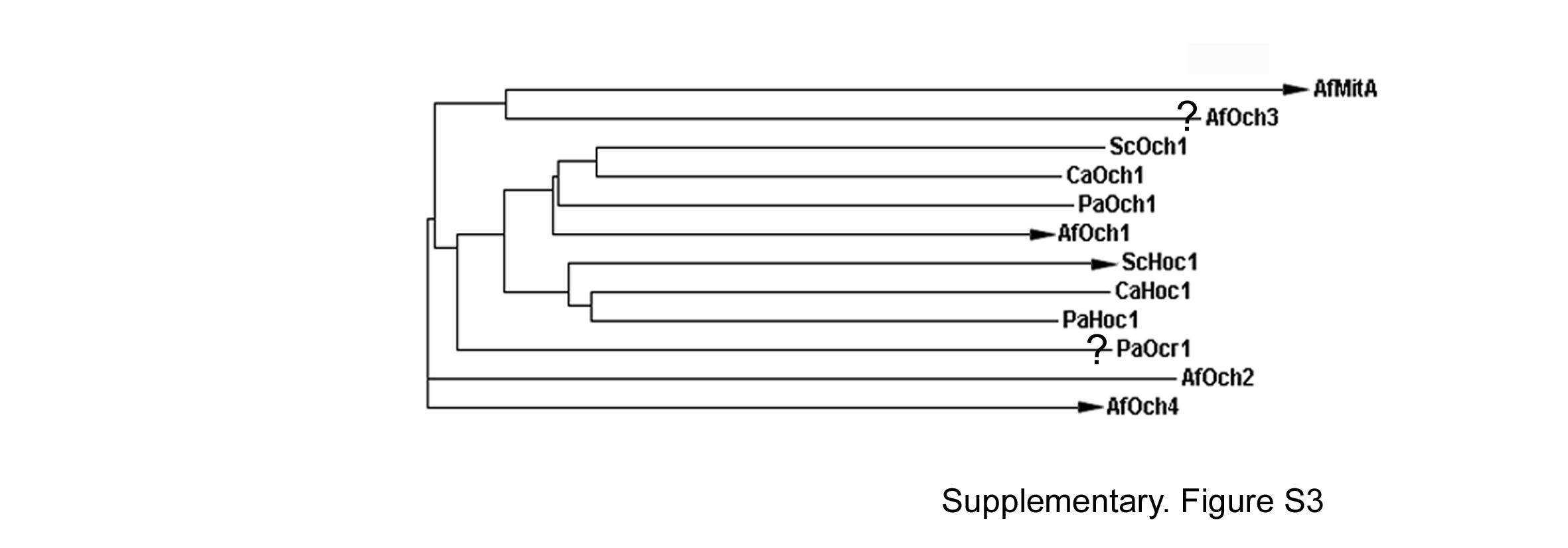

Supplement: Figure S3 — Probability of N-terminal signal sequences and membrane anchors of selected proteins of the Och1 family from A. fumigatus , S. cerevisiae , C. albicans and P. angusta. The sequences were analyzed using the SignalP 3.0 algorithm. The accession numbers of probabilities for a signal sequence or a membrane anchor is given in Panel A. A phylogenetic tree derived from these sequences using ClustalW2 is shown in Panel B. Arrowheads indicate the prediction of a signal peptide, blunt ends indicate a predicted N-terminal membrane anchor. Uncertain predictions are indicated by a question mark. (TIF) [file pone.0015729.s003.tif]

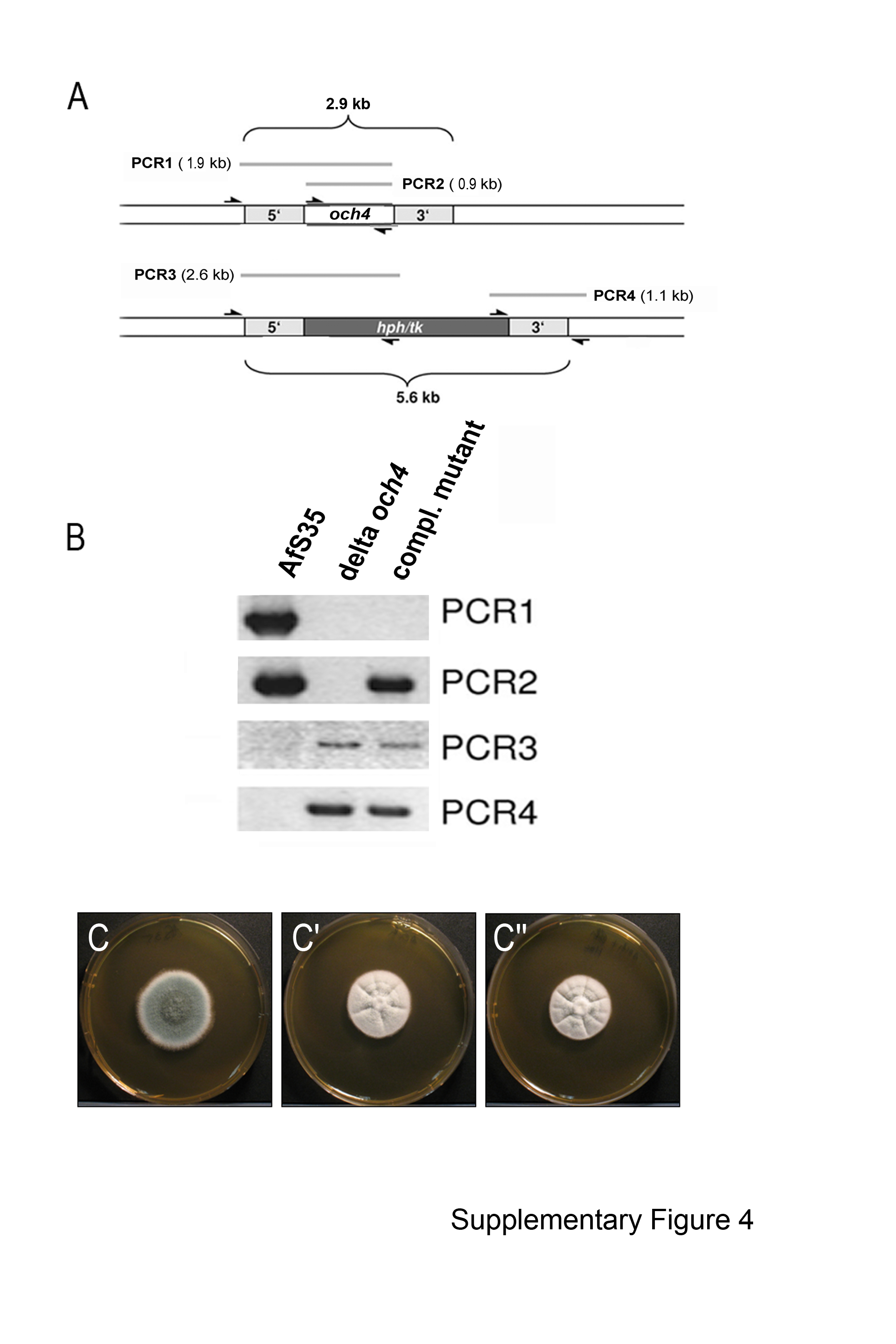

Supplement: Figure S4 — Construction of the Δ afoch4 mutant and its complementation. (A) Structure of the genomic afoch4 gene and the deleted afoch1::hph/tk locus. Approximately 1 kb of the 5′ and 3′ regions of afoch4 (gray boxed areas) were used for construction of the deletion cassette. The positions of the primers used for PCR amplifications and the resulting PCR products (PCR 1–4) are indicated. (B) Equal amounts of genomic DNA of AfS35, Δafocht4 and Δafoch4 + afoch4 were used as template for PCR amplification of the regions indicated in panel A (PCR 1–4). (C) The afoch4 gene is unable to restore wild type sporulation in a Δafocht1 mutant. Colonies grown on Sabouraud medium +100 mM CaCl2 at 37°C are shown for the parental strain AfS35 (C), the Δafoch1 mutant (C′) and the Δafoch1 + afoch4 strain. (TIF) [file pone.0015729.s004.tif]
