# Supplementary material for: Approaching the Secrets of N-Glycosylation in Aspergillus fumigatus: Characterization of the AfOch1 Protein
Source: PLoS One. 2010 Dec 29;5(12):e15729. doi: 10.1371/journal.pone.0015729 (PMC3012087; doi:10.1371/journal.pone.0015729)
Supplement: Table S1 — Probability of N-terminal signal sequences and membrane anchors of Och1 proteins. The Och1 sequences of the indicated fungal species were analyzed using the SignalP 3.0 algorithm. The accession numbers of the analyzed sequences and their homology to the reference ScOch1 protein sequence are given. (DOC) [file pone.0015729.s005.doc]

**Supplementary Table 1:**

| species | Acc. number | signal peptide | signal anchor | homology to ScOch1 (identity/similarity in %) |
| --- | --- | --- | --- | --- |
| *Aspergillus fumigatus* | AFUA_5G08580 | 0.961 | 0.039 | 33.7 / 43.3 |
| *Aspergillus niger* | CAK39413 | 0.972 | 0.028 | 33.3 /44.2 |
| *Aspergillus nidulans* | AN4716.4 | 0.943 | 0.056 | 30.9 / 45.4 |
| *Neurospora crassa* | EAA36513 | 0.964 | 0.036 | 31.0 / 45.5 |
| *Magnaporte grisea* | EAQ71210 | 0.972 | 0.027 | 29.7 / 42.3 |
| *Ashbya gossypii* | AAS53836 | 0.897 | 0.102 | 44.7 / 61.6 |
| *Histoplasma capsulata* | HCEG_06474 | 0.972 | 0.028 | 30.2 / 42.1 |
| *Saccharomyces cerevisiae* | YGL038C | 0.067 | 0.929 | 100 / 100 |
| *Schizosaccharomyces pombe* | CAD24818 | 0.156 | 0.840 | 27.1 / 41.6 |
| *Candida albicans* | orf19.7391 | 0.031 | 0.965 | 37.3 / 51.3 |
| *Cryptococcus neoformans* | CNBG_1711.2 | 0.002 | 0.998 | 19.8 / 31.9 |
| *Pichia angusta* (*Hansenula polymorpha*) | AAS77488 | 0.329 | 0.663 | 36.1 / 52.3 |
| *Pichia pastoris* | XP_002489596 | 0.003 | 0.927 | 35.0 / 47.9 |
