# Supplementary material for: Approaching the Secrets of N-Glycosylation in Aspergillus fumigatus: Characterization of the AfOch1 Protein
Source: PLoS One. 2010 Dec 29;5(12):e15729. doi: 10.1371/journal.pone.0015729 (PMC3012087; doi:10.1371/journal.pone.0015729)
Supplement: Table S2 — Probability of N-terminal signal sequences and membrane anchors for members of the Och1 family of A. fumigatus , S. cerevisiae , C. albicans , and P. augusta . The Och1 sequences of the indicated fungal species were analyzed using the SignalP 3.0 algorithm. The probabilities of N-terminal signal peptides and membrane anchors, as well as the homologies to the reference ScOch1 protein sequence are given. (DOC) [file pone.0015729.s006.doc]

**Supplementary Table 2**

| species |  | signal peptide | signal anchor |
| --- | --- | --- | --- |
| *Aspergillus fumigatus* | AfOch1 | 0.961 | 0.039 |
| *Aspergillus fumigatus* | AfOch2 | 0.049 | 0.938 |
| *Aspergillus fumigatus* | AfOch3 | 0.600 | 0.397 |
| *Aspergillus fumigatus* | AfOch4 | 0.852 | 0.112 |
| *Asperillus fumigatus* | AfMitA | 0.985 | 0.013 |
| *Saccharomyces cerevisiae* | ScOch1 | 0.067 | 0.929 |
| *Saccharomyces cerevisiae* | ScHoc1 | 0.856 | 0.144 |
| *Candida albicans* | CaOch1 | 0.031 | 0.965 |
| *Candida albicans* | CaHoc1 | 0.003 | 0.997 |
| *Pichia angusta* (*Hansenula polymorpha*) | PaOch1 | 0.329 | 0.663 |
| *Pichia angusta* (*Hansenula polymorpha*) | PaHoc1 | 0.364 | 0.629 |
| *Pichia angusta* (*Hansenula polymorpha*) | PaOcr1 | 0.620 | 0.373 |
