# Supplementary material for: Approaching the Secrets of N-Glycosylation in Aspergillus fumigatus: Characterization of the AfOch1 Protein
Source: PLoS One. 2010 Dec 29;5(12):e15729. doi: 10.1371/journal.pone.0015729 (PMC3012087; doi:10.1371/journal.pone.0015729)
Supplement: Table S3 — Oligonucleotides used in this study. (DOC) [file pone.0015729.s007.doc]

**Supplementary Table 3**

| Och1-5’ | ATGCTCACCTTCAGGAAGTCG |
| --- | --- |
| Och1-3’ | TTAGTGGACTTGGTCTTTAGT |
| Och1-3’-b | GCACCACTAAAGTCGTGTTTCAC |
| Och1-upstream-5’ | gggcggccgcatctgagtcataaaatgtcc |
| Och1-upstream-3’ | GGGCCTGAGTGGCCAAGAAGAATCACAGTAGCGC |
| Och1-downstream-5’ | gggccatctaggccgacacagtgctttccatcaaaa |
| Och1-downstream-3’ | GGGCGGCCGCTTCCAACGGGACACCAGTCC |
| trpCt-fwd | CAGAATGCACAGGTACACTTG |
| hph-3-SmaI | TCCCGGGCTATTCCTTTGCCCTCGGACGAG |
| Och1-cast-5’ | GCCACCTGATGGTTAAGAAACG |
| Och1-3’UTR-rev | GACTATCCCGACGTAGACGTG |
| Och1+TMD-5’ | AGTTCGATTAAGATCCCCGCCGTTCTTAACACGGAA |
| Och1+TMD-3’ | GCGAGAGATTAAGAAAAGGAGGACGACGAATGTAAT |
| Och4-5’ | ATGCGGCCTTTCGTTGCCATC |
| Och4-3’ | CTAACACAGCCATGGTAGGCT |
| Och4-upstream-5’ | GACGAACGCACTATCAGGCAA |
| Och4-upstream-3’ | ATCGATGGCCTGAGTGGCCCGGTGTCGCGCTCCACAGATC |
| Och4-downstream-5’ | ATCGATGGCCATCTAGGCCCATTCTTTGTTATTAGCGTTA |
| Och4-downstream-3’ | ACATACATAACCTGATCGGC |
| Och4-cast-5’ | CGTTGCACGATTCCACAACGG |
| Och4-3’UTR-rev | TAGGCGGCTGGTTACTGTGTT |

Oligonucleotides used in this study.
